# Blue-green spaces, heat stress, and health-supportive public-space use under urban stress: machine learning and SEM evidence from jogging flow in Fuzhou, China

**DOI:** 10.3389/fpubh.2026.1870274

**Published:** 2026-06-16

**Authors:** Fan Zhang, Gwon-Soo Bahn, Hongxu Peng, Tianyin Jiang, Jian Sun

**Affiliations:** 1 School of Fine Art and Design, Guizhou Education University, Guizhou, China; 2 Department of Landscape Architecture, Dong-A University, Busan, Republic of Korea; 3 School of Architecture and Urban-Rural Planning, Fuzhou University, Fuzhou, China; 4 Academy of Art Design, Fujian Business University, Fuzhou, China; 5 Xiamen Academy of Arts and Design, Fuzhou University, Xiamen, China

**Keywords:** blue-green spaces, community resilience, health-supportive public-space use, heat stress, jogging flow, machine learning, public activity opportunities, urban pressure

## Abstract

Urban blue-green spaces (BGS) are increasingly discussed in relation to health-oriented and resilience-oriented urban planning, particularly in high-density cities exposed to heat-related environmental stress. However, empirical evidence remains limited on how BGS and heat stress are associated with everyday public-space use. Using Fuzhou, China, as a case study, we integrated crowdsourced jogging data, BGS indicators, land surface temperature (LST), and urban development variables to analyze jogging flow as a behavioral proxy for health-supportive public-space use. Machine learning (ML) and structural equation modeling (SEM) were combined to identify key environmental correlates and examine their direct and indirect pathways. Nine ML models were compared, and SHAP analysis was used to interpret feature importance and nonlinear relationships. The results show that distance to vegetation (DTP), green view index (GVI), sky view index (SVI), and LST were key factors associated with jogging flow. BGS-related variables showed positive associations with jogging flow, whereas higher LST was associated with lower predicted jogging flow. SEM further indicated that green space, blue space, and public activity opportunity proxies were positively associated with jogging flow, while heat stress showed a negative direct association. These findings suggest that BGS may be linked to health-supportive public-space use by providing favorable environmental and activity-related conditions. The study provides planning-relevant evidence for understanding how blue-green environments may contribute to public-space vitality and resilience-oriented urban planning under heat-related urban stress.

## 1 Introduction

High-density cities are increasingly exposed to heat-related environmental stress, intensive land development, and growing public-health demands. These pressures may affect residents’ willingness and ability to use urban public spaces for daily physical activity, especially in hot and humid urban environments. Maintaining accessible, comfortable, and activity-supportive public spaces has therefore become an important issue for health-oriented and resilience-oriented urban planning. Outdoor jogging provides a useful behavioral lens for examining this issue because it is a low-cost, routine, and spatially traceable form of physical activity. Spatial variations in jogging flow can reflect how residents use urban public spaces for everyday health-supportive activities under different environmental conditions (1–3). This raises an important question of what kinds of urban environmental conditions are associated with more active public-space use under heat-related urban stress.

Among these conditions, urban blue-green spaces (BGS) are particularly relevant because they combine environmental regulation, visual quality, and recreational functions. BGS, including vegetation, parks, waterfronts, rivers, and other natural or semi-natural environments, can provide thermal comfort, visual attractiveness, psychological restoration, and opportunities for outdoor recreation (4–6). Related sponge-city and low-impact development studies also suggest that blue-green infrastructure can generate multi-objective environmental benefits, such as runoff control, pollutant reduction, and stormwater utilization, thereby strengthening its relevance to resilience-oriented urban planning (7). In crisis or stress contexts, such as extreme heat, public health emergencies, or high-density urban pressure, BGS may help residents maintain daily activity routines and reduce psychological burden (8–10). Therefore, understanding how BGS are associated with public-space use is relevant to health-oriented and resilience-oriented urban planning.

In this study, jogging flow refers to the spatial intensity of recorded jogging behavior derived from crowdsourced running records. It is treated as a behavioral proxy for health-supportive public-space use rather than merely as an exercise indicator. Areas with higher jogging flow may indicate stronger public-space attractiveness, better environmental support for physical activity, and more favorable spatial conditions for informal public encounters (11–14).

A growing body of empirical research has examined the relationships between blue-green spaces, built-environment characteristics, and outdoor physical activity. Studies on green spaces and street greenery have shown that park accessibility, green-view exposure, and visually comfortable streetscapes are closely associated with walking, jogging, and other active behaviors (6, 15–17). Related studies on blue spaces and waterfront environments have also suggested that rivers, lakes, and waterfront greenways can provide attractive routes and recreational settings for running and cycling activities (18). In addition to blue-green environmental quality, thermal conditions and urban development intensity have received increasing attention. Higher heat exposure may reduce the attractiveness and usability of outdoor spaces for physical activity, particularly in hot and humid cities (19, 20). Meanwhile, dense road networks, commercial intensity, residential density, and access to sports facilities may be associated with jogging behavior through route continuity, accessibility, safety, and activity-related spatial opportunities (11–13, 21).

Although these studies provide important evidence on the environmental correlates of outdoor physical activity, three gaps remain. First, previous research has often examined green space, blue space, thermal environment, and built-environment factors separately, making it difficult to clarify how these dimensions are jointly associated with jogging flow. Second, many studies emphasize the direct associations between individual environmental variables and physical activity. Less attention has been paid to possible indirect pathways linking blue-green spaces, public activity opportunity proxies, urban pressure, heat stress, and jogging flow. Third, the relationships between urban environmental conditions and jogging flow may be nonlinear and interactive, but these complex patterns are not always integrated with pathway-based explanation. Therefore, an analytical framework that combines interpretable machine learning with SEM can help identify key environmental correlates while also examining how these factors are linked within a broader pathway structure.

To address these gaps, using Fuzhou, China, as a case study, this study investigates how urban BGS, heat stress, and urban environmental conditions are associated with jogging flow. Here, jogging flow is used as a behavioral indicator of health-supportive public-space use under heat-related urban stress. Specifically, this study has three objectives. First, it compares multiple ML models to identify the best-performing model for predicting jogging flow. Second, it uses interpretable ML to identify key BGS, heat-stress, and urban factors associated with jogging flow and to examine their nonlinear and interactive relationships. Third, it applies SEM to explore the direct and indirect associations linking BGS, public activity opportunity proxies, urban pressure, heat stress, and jogging flow. By integrating crowdsourced data, interpretable ML, and pathway analysis, this study provides empirical evidence relevant to resilience-oriented public-space planning in high-density urban contexts.

## 2 Materials and methods

### 2.1 Conceptual framework and analytical workflow

To align the empirical analysis with the resilience-oriented perspective of the special issue while avoiding an overextension of the available data, this study reorganized the original environmental and urban variables into a conceptual framework linking blue-green spaces, public activity opportunity proxies, urban pressure, heat stress, and jogging flow. In this framework, urban blue-green spaces are not treated as direct measures of community resilience. Instead, they are examined as environmental conditions that may be associated with health-supportive public-space use under heat-related urban stress.

The variables were grouped into four conceptual dimensions. First, blue-green space indicators were used to represent the availability, accessibility, and visual quality of blue-green environments. Second, public activity opportunity proxies were represented by spatial variables, including points of interest, accessibility to sports facilities, road density, and residential building density. Because this study does not directly measure social cohesion, social capital, or actual interpersonal interaction, these variables were interpreted only as spatial conditions that may provide opportunities for public activity and informal encounters. Third, urban pressure was represented by socioeconomic, density-related, terrain, and built-environment indicators, reflecting the broader urban context in which blue-green spaces and jogging flow are embedded. Finally, jogging flow was used as the outcome variable and interpreted as a behavioral proxy for health-supportive public-space use.

**Figure 1:**
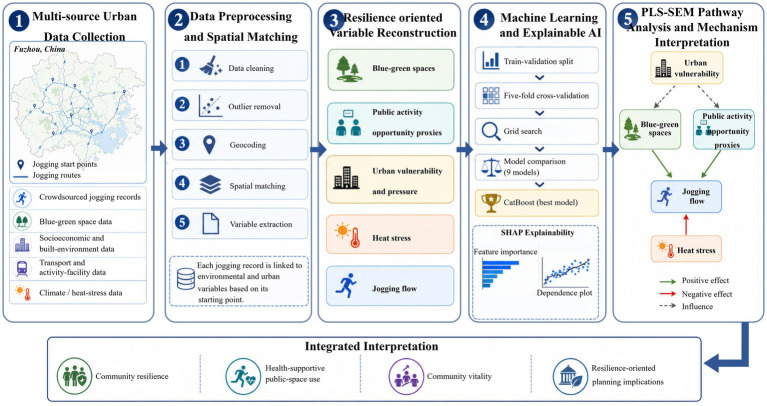
Analytical workflow of the study.

Based on this conceptual framework, the empirical analysis followed four main steps (Figure 1). First, multi-source data were collected and spatially matched to jogging starting points, including crowdsourced jogging records, blue-green space indicators, heat-stress variables, socioeconomic indicators, transportation variables, urban morphology variables, and land-use variables. Second, the variables were organized into the conceptual dimensions of blue-green spaces, public activity opportunity proxies, urban pressure, heat stress, and jogging flow. Third, nine machine-learning models were compared, and the best-performing model was interpreted using SHAP to identify key predictors and nonlinear interaction patterns. Fourth, PLS-SEM was used to examine possible direct and indirect association pathways among the main constructs. This framework guides the subsequent ML and SEM analyses while clarifying that the study offers planning-relevant evidence rather than a direct measurement of community resilience.

### 2.2 Study area

Fuzhou, commonly known as “Rong,” nicknamed Rongcheng, and historically referred to as Mindu, is located in the central-eastern part of Fujian Province (25°15′–26°39′N, 118°08′–120°31′E). The city covers a total area of 11,968.53 km^2^ and has a permanent population of 8.501 million. As a major regional center in southeastern China, Fuzhou has experienced rapid urbanization and increasing development intensity in recent decades, making it a representative case for examining urban environmental conditions under high-density and high-pressure contexts (22).

The city has a typical subtropical monsoon climate, characterized by high temperature, high humidity, and abundant rainfall. These climatic conditions may impose environmental stress on outdoor activities, particularly during hot and humid seasons. In such contexts, the availability and accessibility of blue-green spaces become particularly important for maintaining residents’ daily physical activity and public-space use. Fuzhou is rich in water resources, with substantial locally generated and inflow water volumes, and contains an extensive network of rivers, lakes, and waterfront environments. In addition, the city includes over 1,000 parks with a total green space area of 5,577.3 hectares. The coexistence of abundant blue and green spaces provides diverse environmental conditions for outdoor activities such as jogging, while also offering potential support for mitigating environmental stress and enhancing urban livability.

**Figure 2:**
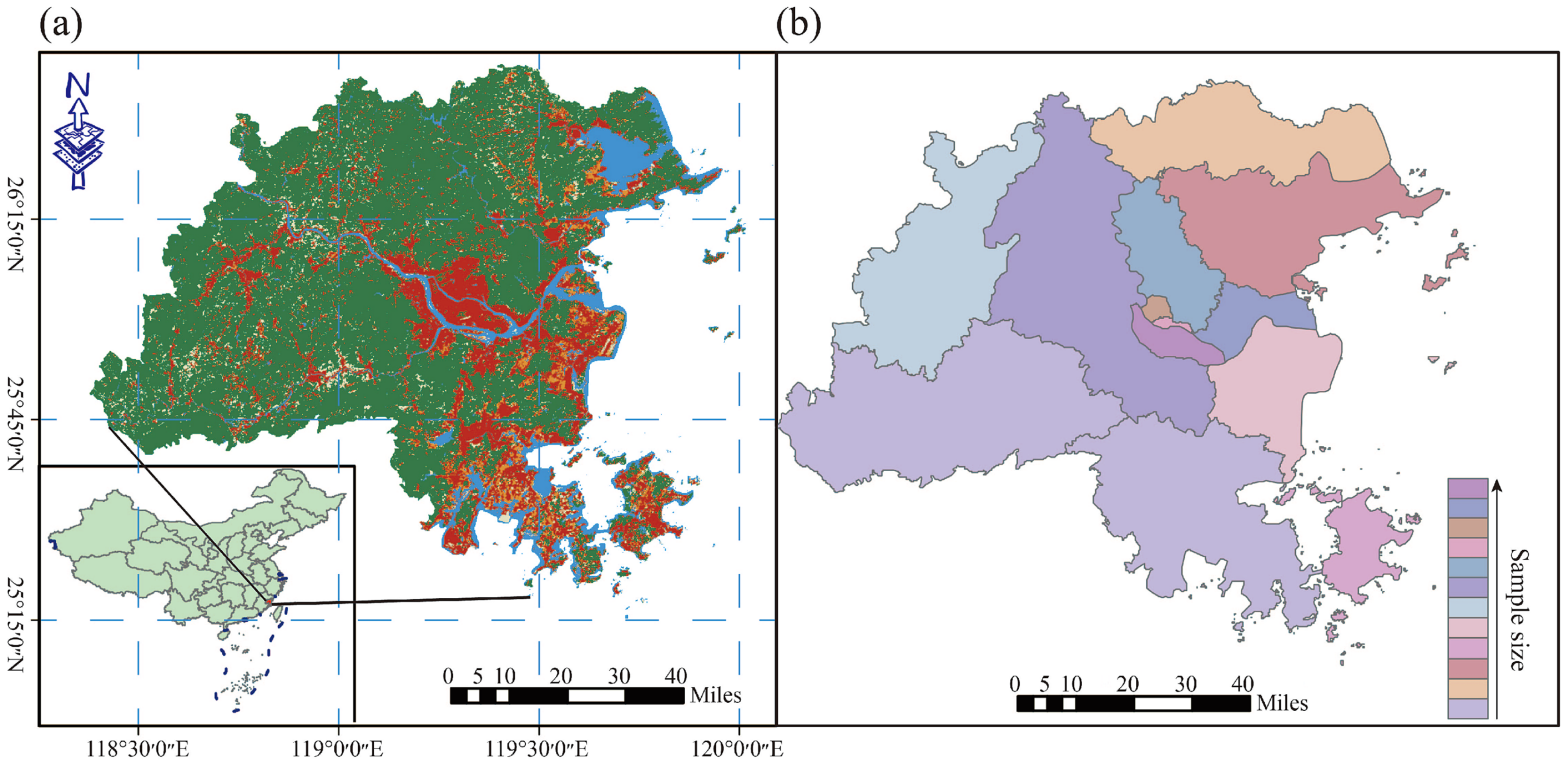
Overview of the study area. **(a)** Study area; **(b)** sample size by county-level administrative unit within the study area.

Administratively, Fuzhou administers six districts, one county-level city, and six counties, and the study area covers the Fuzhou municipal region. The valid jogging records retained after preprocessing were distributed across 10 county-level administrative units within this region. The spatial heterogeneity in urban development, population density, and blue-green space distribution across these administrative units makes Fuzhou a suitable case for examining how blue-green environments, urban pressure, and public-space use are interconnected. Therefore, Fuzhou provides an appropriate empirical setting for examining how blue-green spaces are associated with health-supportive public-space use under high-density urban conditions (Figure 2).

### 2.3 Data sources and preprocessing

#### 2.3.1 Jogging flow data

The dependent variable in this study was jogging flow (JT). Jogging data were obtained from Keep, a widely used Chinese fitness-tracking application. Similar crowdsourced fitness-app and trajectory data have been increasingly used in physical activity–built environment research (23). The jogging records used in this study were collected in September 2024. Python 3.13.5 was used to collect anonymized jogging records, including user ID, jogging duration, speed, distance, and starting latitude and longitude. Because physical activity spaces may extend beyond residential neighborhoods, georeferenced activity records can help capture actual outdoor activity locations more directly than fixed residential buffers (24). In this study, each preprocessed jogging record was treated as one observational sample, and its starting point was used as the spatial reference for linking environmental and socioeconomic variables. This choice was made because starting points were consistently available across all records, whereas complete trajectories may be incomplete, unevenly recorded, or unavailable for part of the crowdsourced dataset.

Data preprocessing was conducted before spatial analysis. The dataset was checked for duplicate records based on anonymized user ID, starting location, date, and duration, and no duplicated records were retained in the final dataset. To improve data reliability, abnormal records were removed. Specifically, samples with jogging distances shorter than 400 m or longer than 50 km were excluded. Records with average jogging speeds lower than 5 km/h or higher than 25 km/h were also removed. In addition, data points located more than 30 km from sports facilities, water bodies, or green spaces were discarded. After preprocessing, 20,207 valid jogging records were retained for subsequent analysis and were distributed across 10 county-level administrative units in Fuzhou.

Because Keep is a crowdsourced fitness-tracking platform, the retained records may overrepresent active, health-conscious, and mobile-app users. Therefore, the dataset was used to capture the spatial behavior of recorded jogging-app users rather than to represent the full population of Fuzhou. In addition, the use of jogging starting points does not measure the full environmental exposure experienced along complete jogging routes. Instead, it captures the spatial distribution of jogging origins and their surrounding environmental conditions. Therefore, jogging flow should be interpreted as a starting-point-based proxy for health-supportive public-space use rather than a complete route-level exposure measure.

#### 2.3.2 Environmental and urban variables

To examine multidimensional urban correlates of jogging flow, this study included 38 independent variables covering climate, air quality, blue space, green space, socioeconomic conditions, transportation infrastructure, environmental ecology, public activity opportunity proxies, urban morphology, land use, and heat stress. Detailed variable names, abbreviations, means, and units are reported in Table 1. These variables were subsequently reorganized into the conceptual dimensions described in Section 2.1 for the SEM analysis. In particular, POI, DTS, Road_D, and RBD were used as proxies for public activity opportunities rather than direct measures of social cohesion or social capital. The selection of blue- and green-space indicators was also consistent with recent efforts to construct multidimensional blue-green space evaluation systems and improve urban blue-space mapping using open geospatial datasets (25, 26). Because the jogging records were collected in September 2024, time-varying environmental variables, including meteorological, air-quality, LST, NDVI, and high-temperature-day indicators, were matched to September 2024. For relatively stable spatial variables, such as blue-green space patches, POI, road networks, building morphology, DEM, and land use, the closest available data version or access date was used. The main data sources, temporal coverage or data versions, and spatial resolutions are summarized in Table 2.

**Table 1:** Variables.

Group	Category	Variable	Mean	Unit
Dependent variables	Jogging flow	JT	1.97	unitless
Independent variables	Blue space	DTW	0.37	km
		SVI	0.27	unitless
		Blue_sp	278.86	count
		Water_Area	37.2	km^2^
	Green space	GVI	0.13	unitless
		Green_Area	385.87	km^2^
		Green_sp	769.2	count
		DTP	2.23	km
	Heat stress	LST	28.6	°C
		High_temp_day	0.247	unitless
	Public activity opportunity proxies	POI	166.33	count
		DTS	1.15	km
	Climate	TEMP	27.09	°C
		WS	7.03	km/h
		PREP	171.63	mm
	Air quality	AQI	21.79	unitless
		PM_2.5_	18.33	μg/m^3^
		NO_2_	14.47	μg/m^3^
		O_3_	72.01	μg/m^3^
		PM_10_	62.47	μg/m^3^
	Socioeconomic and population	POP_D	7685.71	people/km^2^
		GDP	1207.69	10^8^CNY
		HP	16884.02	CNY/m^2^
	Transportation infrastructure	PTSD	418.71	count
		Subway_N	15.99	count
		Road_L	279.18	km
		Road_D	0.55	km/km^2^
		RI	1371.01	count
	Urban morphology	ADA	585.58	km^2^
		FAR	2.2	unitless
		RBD	0.16	1/km^2^
		Building_Area	125.11	km^2^
	Environmental ecology	NDVI	0.59	unitless
		RL	224.58	km
		PD	11.76	count
		DEM	27.34	m
	Land use	Shannon	0.97	unitless
		Land use mix	0.95	unitless

**Table 2:** Data sources and basic information for variables used in this study.

Variable category and variables	Dataset, source, and platform	Temporal coverage or data version	Spatial resolution or scale
Jogging flow: JT	Anonymized jogging records from the Keep fitness-tracking platform, collected with Python 3.13.5	Sep-24	GPS starting-point records
Climate: TEMP, WS, PREP	Historical weather data from Tianqi24	Sep-24	City- or station-level meteorological records
Air quality: AQI (comprehensive air-quality index), PM₂.₅, NO₂, O₃, PM₁₀	Monthly Urban Air Quality Status for Fujian Province released by the Fujian Provincial Department of Ecology and Environment	Sep-24	City-level monthly air-quality records for Fuzhou
Blue space: DTW, Blue_sp., Water_Area	Esri 10 m Annual Land Cover, accessed through Esri Land Cover Explorer in ArcGIS Living Atlas; the water class was extracted as blue-space data	2024 annual product	10 m raster
Green space: DTP, Green_Area, Green_sp	Esri 10 m Annual Land Cover, accessed through Esri Land Cover Explorer in ArcGIS Living Atlas; green-space patches were extracted from vegetation-related land-cover classes	2024 annual product	10 m raster
Street-level visual indicators: GVI, SVI	Baidu Street View images accessed through Baidu Maps; DeepLabv3 + trained on the Cityscapes dataset was used for semantic segmentation	Accessed in September 2024	Street-view images matched to the surrounding area of jogging starting points
Heat stress: LST	MODIS Terra Land Surface Temperature and Emissivity 8-Day L3 Global 1 km SIN Grid Version 6.1 (MOD11A2), accessed through Google Earth Engine	Sep-24	1 km raster; 8-day composite
Heat stress: High_temp_day	ERA5-Land Daily Aggregated dataset, 2 m air temperature, Copernicus Climate Change Service Climate Data Store	Sep-24	ERA5-Land reanalysis grid; daily aggregated air temperature
Public activity opportunity proxies: POI, DTS	OpenStreetMap POI and sports-facility data	OpenStreetMap data accessed in 2024	Vector POI records
Socioeconomic and population: POP_D, GDP, HP	Fuzhou Statistical Yearbook 2024, Fuzhou Municipal People’s Government	2024 Statistical Yearbook	District-level, county-level, or corresponding statistical-unit records
Transportation infrastructure: PTSD, Subway_N, Road_L, Road_D, RI	OpenStreetMap road-network and public-transport features, accessed through Geofabrik and OpenStreetMap	OpenStreetMap data accessed in 2024	Vector road, station, and public-transport stop records
Urban morphology: ADA, FAR, RBD, Building_Area	OpenStreetMap building footprints and administrative boundary data, accessed through Geofabrik and OpenStreetMap	OpenStreetMap data accessed in 2024	Vector building footprints and administrative polygons
Environmental ecology: NDVI, DEM	MODIS Terra Vegetation Indices 16-Day L3 Global 250 m SIN Grid product (MOD13Q1, Version 6.1), accessed through NASA Earthdata; DEM from GEBCO_2024 Grid	September 2024 for NDVI; GEBCO_2024 for DEM	250 m raster for NDVI; 15 arc-second grid for DEM
Landscape and land-use indicators: RL, PD, Shannon, Land use mix	Esri 10 m Annual Land Cover, accessed through Esri Land Cover Explorer in ArcGIS Living Atlas	2024 annual product	10 m raster

Meteorological variables, including temperature, wind speed, and precipitation, were obtained from Tianqi24. Air-quality variables, including the comprehensive air-quality index, PM_2.5_, NO_2_, O_3_, and PM_10_, were obtained from the monthly urban air-quality status released by the Fujian Provincial Department of Ecology and Environment. LST was derived from the MODIS MOD11A2 Version 6.1 8-day land surface temperature product through Google Earth Engine, while High_temp_day was calculated from ERA5-Land daily 2 m air temperature. NDVI was derived from the MODIS/Terra Vegetation Indices 16-Day L3 Global 250 m SIN Grid product (MOD13Q1, Version 6.1) through NASA Earthdata. DEM data were obtained from the GEBCO_2024 Grid. Blue-space, green-space, and land-use indicators were derived from the Esri 10 m Annual Land Cover product. Specifically, the water class was extracted to calculate DTW, Blue_sp., and Water_Area, while vegetation-related land-cover classes were extracted to calculate DTP, Green_Area, and Green_sp. Land-cover classes were also reclassified to calculate the Shannon index and land use mix. Street-level visual indicators, including GVI and SVI, were derived from Baidu Street View images accessed through Baidu Maps. DeepLabv3+ trained on the Cityscapes dataset was used for semantic segmentation to extract vegetation and sky pixels, and the resulting visual indicators were matched to the surrounding area of jogging starting points.

Socioeconomic and population variables, including POP_D, GDP, and HP, were obtained from the Fuzhou Statistical Yearbook 2024. POI and sports-facility data were obtained from OpenStreetMap and were used to represent the spatial distribution of activity-related destinations. Transportation infrastructure variables, including PTSD, Subway_N, Road_L, Road_D, and RI, were derived from OpenStreetMap road-network and public-transport features accessed through Geofabrik/OpenStreetMap. Urban morphology variables, including ADA, FAR, RBD, and Building_Area, were derived from OpenStreetMap building footprints and administrative boundary data.

According to the data type, the variables were processed using distance calculation, density estimation, area statistics, raster aggregation, semantic segmentation, or spatial matching. Distance-based variables, such as DTP, DTW, and DTS, were calculated as the distance from each jogging starting point to the nearest corresponding spatial feature. Count- and density-based variables, such as POI, Road_D, RBD, Green_sp., and Blue_sp., were calculated within the corresponding analytical units. Raster-based indicators, including LST, NDVI, DEM, and land-cover-derived variables, were clipped to the Fuzhou study area and aggregated to the analytical units. Finally, all variables were spatially matched to jogging starting points to construct the modeling dataset.

#### 2.3.3 Data preprocessing and spatial matching

All spatial variables were processed in a unified coordinate reference system before spatial matching. Jogging records with invalid coordinates, abnormal distance, abnormal speed, or locations outside the study area were removed. Distance-based variables, including DTP, DTW, and DTS, were calculated as the Euclidean or network distance from each jogging starting point to the nearest vegetation patch, water body, and sports facility, respectively. Count- and density-based variables, including POI, public transport stops, road intersections, road density, residential building density, blue-space counts, and green-space counts, were calculated within buffers around each jogging starting point. Area-based variables, including green area, water area, built-up area, and administrative district area, were calculated at the spatial unit level and assigned to jogging records according to their starting-point locations.

The starting point of each jogging record was used as the spatial matching unit for three reasons. First, starting points were consistently available across all valid records and therefore provided a unified spatial reference for linking jogging observations with multi-source environmental variables. Second, starting locations often correspond to the neighborhood-scale public-space environment from which recorded jogging flow originates, making them relevant for examining the spatial conditions associated with jogging-flow origins. Third, using starting points helped avoid inconsistencies caused by incomplete, uneven, or heterogeneous trajectory records in crowdsourced fitness-app data. This approach is therefore suitable for analyzing spatial associations between jogging origins and nearby environmental conditions. However, it does not measure full-route environmental exposure. The results should therefore be interpreted as associations between jogging-flow origins and surrounding blue-green, heat-stress, and urban conditions, rather than as evidence of environmental exposure along complete jogging routes.

After all variables were matched to jogging records, the final dataset was used for multicollinearity testing, machine-learning modeling, SHAP interpretation, and SEM analysis. The detailed variable-screening and modeling procedures are described in Sections 2.4 and 2.5.

### 2.4 Machine learning model approach and SHAP interpretation

To comprehensively evaluate the performance of different machine-learning algorithms in predicting jogging flow, this study selected nine models, including RF, ExtraTrees, XGBoost, LightGBM, CatBoost, AdaBoost, MLP, SVR, and DT. After outlier removal, the dataset was randomly divided into training and validation sets at a ratio of 7:3. Grid search combined with five-fold cross-validation was used to tune the hyperparameters of the nine algorithms (Table 3). To avoid selecting predictors only on the basis of prior expectations, all 38 independent variables listed in Table 1 were first treated as candidate predictors. Before model training, multicollinearity was assessed using the mctest package in R, and variables with VIF values greater than 5 were excluded. The retained variables were then entered into the nine machine-learning models under the same training and validation procedure. After the optimal model was identified, SHAP values were used to rank the relative contribution of the retained predictors. Because the variable system included multiple categories of environmental and urban indicators, the subsequent interpretation focused on the top 12 SHAP-ranked variables, which were regarded as the main predictors associated with jogging flow in this dataset. Accordingly, the subsequent dependence and interaction analyses focused on variables with higher SHAP contributions. Because the jogging records and environmental variables were spatially referenced, potential spatial autocorrelation was recognized as an important methodological issue. This study focused on predictive associations rather than spatial causal effects; therefore, the results should be interpreted as spatially referenced associations between environmental conditions and jogging flow.

**Table 3:** Parameter settings and optimal parameters of nine machine learning algorithms.

Model	Param Grid	Best_Params
RF	n_estimators: [100, 200, 300, 500, 1,000], max_depth: [1, 3, 5, 7, 9], min_samples_split: [2, 3, 5, 7, 9]	max_depth: 9, min_samples_split: 3, n_estimators: 200
XGBoost	n_estimators: [100, 200, 300, 500, 1,000], max_depth: [1, 3, 5, 7, 9], learning_rate: [0.01, 0.05, 0.1, 0.5, 1]	learning_rate: 0.05, max_depth: 9, n_estimators: 1000
LightGBM	n_estimators: [100, 200, 300, 500, 1,000], max_depth: [1, 3, 5, 7, 9], learning_rate: [0.01, 0.05, 0.1, 0.5, 1]	learning_rate: 0.5, max_depth: 5, n_estimators: 1000
ExtraTrees	n_estimators: [100, 200, 300, 500, 1,000], max_depth: [1, 3, 5, 7, 9], min_samples_split: [2, 3, 5, 7, 9]	max_depth: 9, min_samples_split: 5, n_estimators: 1000
AdaBoost	n_estimators: [100, 200, 300, 500, 1,000], learning_rate: [0.01, 0.05, 0.1, 0.5, 1], estimator: [1, 3, 5, 7, 9]	estimator_max_depth: 9, learning_rate: 1, n_estimators: 100
SVR	C: [0.1, 1, 2, 3, 5], gamma: [0.01, 0.05, 0.1, 0.5, 1]	C: 5, gamma: 1
CatBoost	iterations: [100, 200, 300, 500, 1,000], depth: [1, 3, 5, 7, 9], learning_rate: [0.01, 0.05, 0.1, 0.5, 1]	depth: 9, iterations: 1000, learning_rate: 0.1
DT	max_depth: [1, 3, 5, 7, 9], min_samples_split: [2, 3, 5, 7, 9], min_samples_leaf: [1, 3, 5, 7, 9]	max_depth: 9, min_samples_leaf: 2, min_samples_split: 3
MLP	hidden_layer_sizes: [(50,), (100,), (50, 50), (100, 50)], activation: [‘relu’, ‘tanh’], alpha: [0.0001, 0.001, 0.01], learning_rate_init: [0.01, 0.05, 0.1, 0.5, 1], max_iter: [100, 200, 300, 500, 1,000]	activation: relu, alpha: 0.001, hidden_layer_sizes: (100,), learning_rate_init: 0.05, max_iter: 100

To predict jogging flow and identify key environmental correlates, this study combined comparative machine-learning modeling with SHAP-based model interpretation. SHAP (Shapley Additive Explanations) is a game-theoretic framework that assigns each feature an importance value by estimating its marginal contribution to model predictions (27). It provides both local explanations for individual predictions and global importance measures by aggregating feature contributions across samples (28). Based on the optimal model, SHAP was applied in this study for two purposes: (1) global importance analysis, by calculating the mean absolute SHAP value of each variable to identify the key factors associated with jogging flow; and (2) dependence analysis, by using SHAP dependence plots to examine the nonlinear relationships between key predictors and jogging flow, as well as their interaction effects.

In addition, SHAP dependence and interaction analyses were used to examine how blue-green space variables interacted with urban pressure indicators and public activity opportunity proxies.

### 2.5 PLS-SEM pathway analysis

SEM allows the simultaneous examination of structural and measurement relationships among variables and is widely used to analyze direct and indirect pathways in complex models (29, 30). To examine the main pathways through which the identified factors were associated with jogging flow, this study constructed a partial least squares structural equation model (PLS-SEM) using the plspm package in R (version 4.5.1). PLS-SEM was selected rather than covariance-based SEM for three reasons. First, the purpose of the SEM analysis was primarily exploratory and prediction-oriented, aiming to examine possible pathway associations among blue-green spaces, public activity opportunity proxies, urban pressure, heat stress, and jogging flow, rather than to confirm a well-established theoretical covariance structure. Second, the model included multiple observed indicators and indirect pathways, and PLS-SEM is suitable for estimating relatively complex path models with fewer distributional restrictions. Third, the variables were derived from multi-source urban spatial datasets and crowdsourced jogging records, which may not fully satisfy the multivariate normality assumption commonly required in covariance-based SEM. Therefore, PLS-SEM was considered appropriate for examining direct and indirect associations within the proposed conceptual framework (31, 32).

Following Section 2.1, the PLS-SEM model included blue-green spaces, public activity opportunity proxies, urban pressure, heat stress, and jogging flow. Jogging flow was treated as the dependent construct, while POI, DTS, Road_D, and RBD were used as indicators of public activity opportunity proxies. The model examined direct and indirect pathway associations among these constructs.

Model evaluation focused on explanatory power, path strength, and statistical significance. The goodness-of-fit (GOF) value was used as a descriptive index of overall model adequacy in the PLS-SEM framework. The *R*^2^ value was used to indicate the proportion of variance explained for endogenous constructs, especially jogging flow. Standardized path coefficients were used to describe the direction and relative strength of associations between latent constructs. Bootstrap resampling was used to assess the statistical significance of direct and indirect effects, with *p*-values used to determine whether the estimated pathways were statistically significant (31, 32). The detailed pathway results are reported in Section 3.6.

## 3 Results

### 3.1 Optimal machine learning model

**Figure 3:**
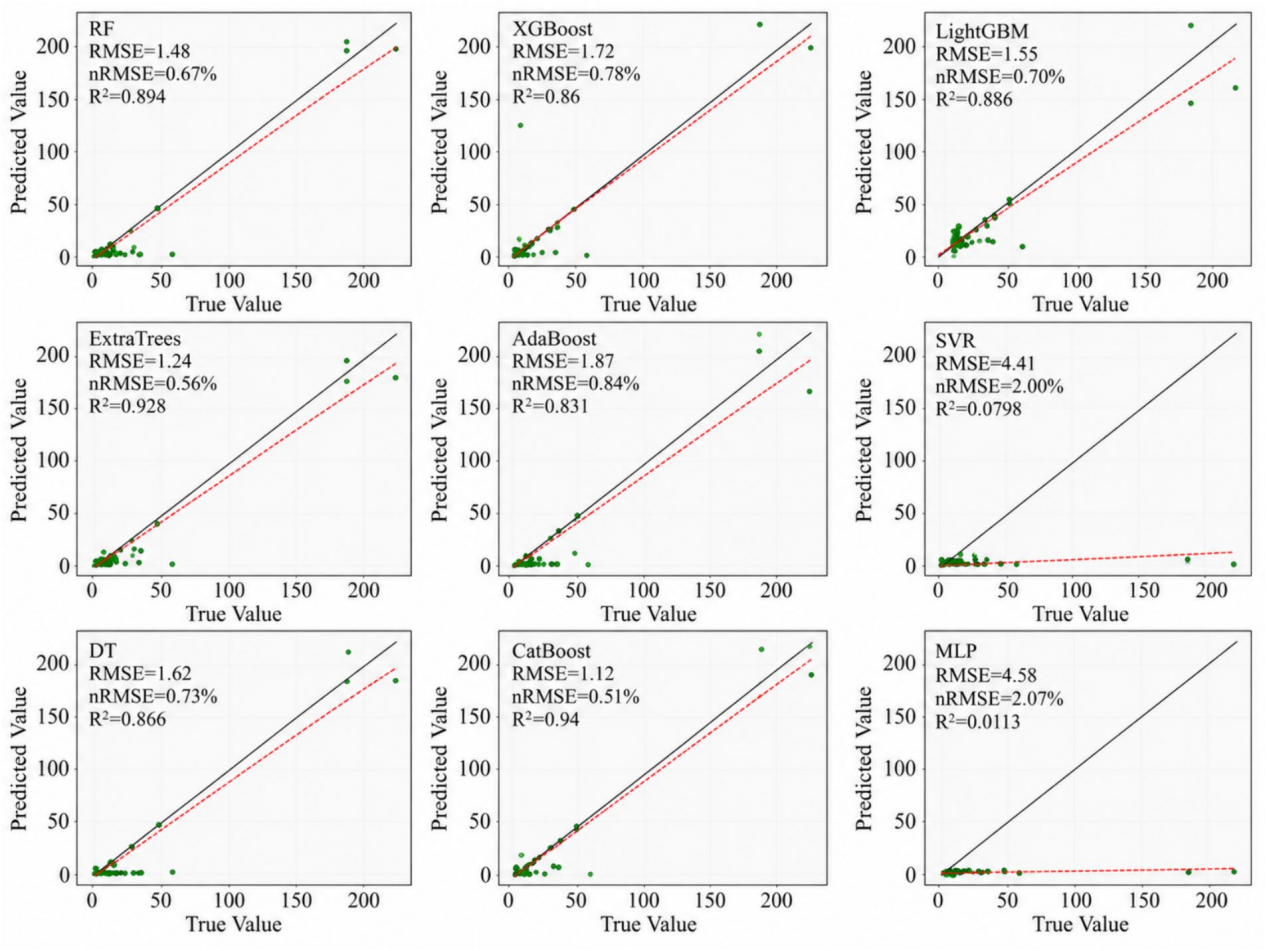
Model fitting performance of nine machine learning algorithms.

To identify the best-performing algorithm for predicting jogging flow, this study used root mean square error (RMSE), coefficient of determination (*R*^2^), and normalized root mean square error (nRMSE) as evaluation metrics (Figure 3). The detailed predictive performance of the nine machine-learning models is further summarized in Table 4 to support a transparent model-selection process for subsequent SHAP interpretation. Among the nine machine learning models, CatBoost achieved the highest *R*^2^ and the lowest RMSE and nRMSE, indicating the best overall predictive performance. Therefore, CatBoost was selected as the optimal model for subsequent SHAP interpretation.

**Table 4:** Predictive performance of the nine machine-learning models.

Model	RMSE↓	nRMSE↓	*R*^2^↑
RF	1.48	0.67%	0.894
XGBoost	1.72	0.78%	0.86
LightGBM	1.55	0.70%	0.886
ExtraTrees	1.24	0.56%	0.928
AdaBoost	1.87	0.84%	0.831
SVR	4.41	2.00%	0.0798
DT	1.62	0.73%	0.866
**CatBoost**	**1.12**	**0.51%**	**0.94**
MLP	4.58	2.07%	0.0113

Bold values indicate the best performance among the nine models for each metric.

To further examine whether a small number of high-influence observations dominated the model results, we conducted a robustness check by removing nine training samples with SHAP values greater than 10 across all variables and retraining the CatBoost model using the same hyperparameters. Because SHAP values are model-derived explanation scores rather than original data measurements, the threshold of 10 was used only as a diagnostic criterion for sensitivity testing. This threshold was selected because it identified a small group of observations with exceptionally high explanatory contributions relative to the remaining samples, allowing us to assess whether the model interpretation was driven by these extreme high-SHAP cases. The independent validation set was kept unchanged. The cleaned model showed stable predictive performance, with *R*^2^ increasing from 0.9403 to 0.9517 and RMSE decreasing from 1.12 to 0.9411. This result suggests that the overall model performance and interpretation were not driven by these few high-SHAP observations.

### 3.2 Key environmental correlates of jogging flow

**Figure 4:**
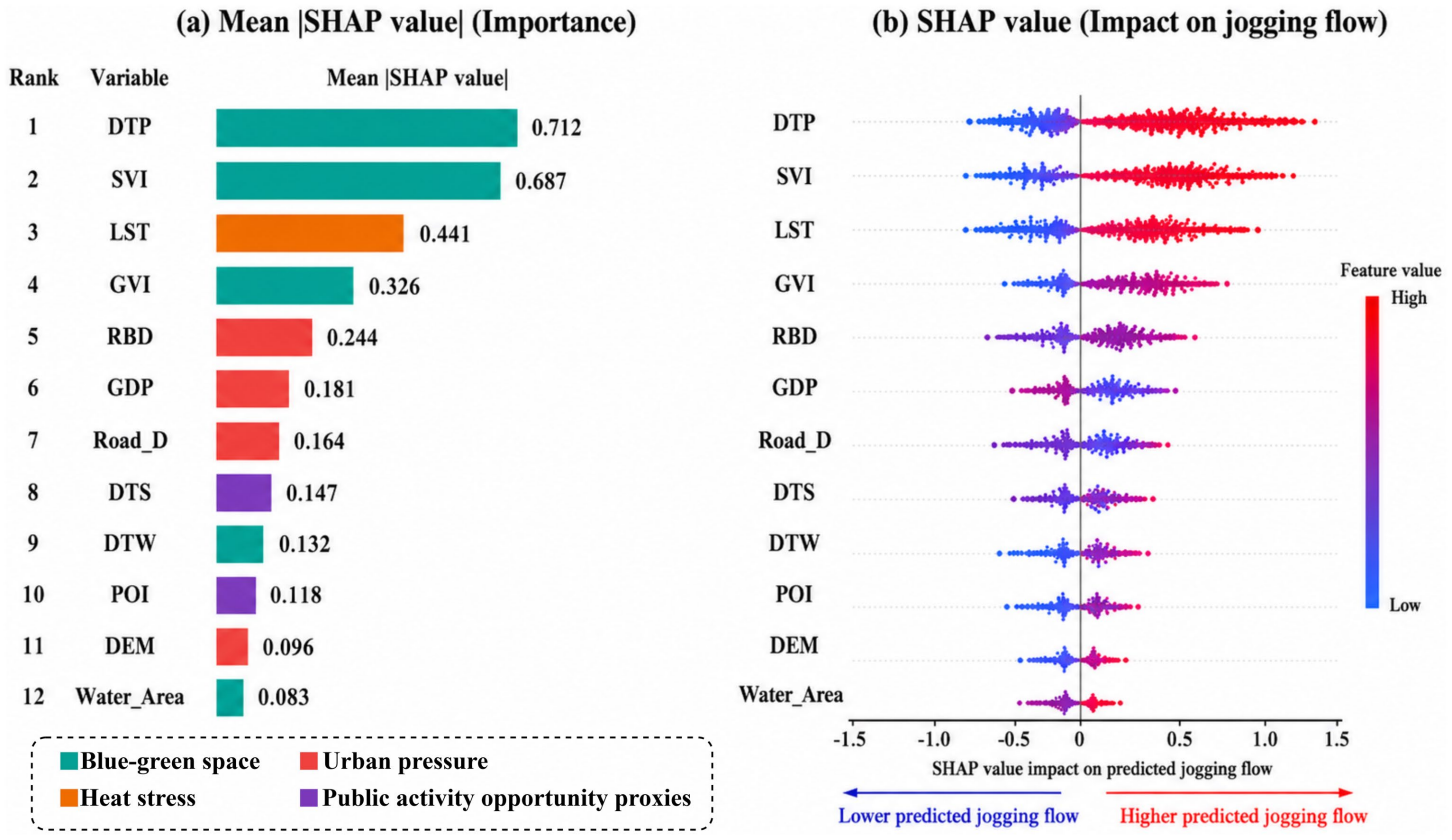
Key environmental correlates of jogging flow: **(a)** Mean absolute SHAP values of the variables, with colors indicating different conceptual dimensions. **(b)** SHAP summary plot showing the direction and distribution of each variable’s contribution to jogging-flow prediction, with colors indicating high and low variable values.

Figure 4 shows the SHAP-based ranking of the retained predictors after variable screening and model selection. DTP and SVI had the highest SHAP contributions, followed by LST, GVI, and RBD, indicating that jogging flow was mainly associated with blue-green space accessibility, visual environmental quality, heat stress, and urban pressure. DTS and POI represented public activity opportunity proxies, whereas GDP, DEM, RBD, and Road_D reflected broader urban pressure conditions. Because the initial predictor set covered multiple dimensions of the urban environment, including air quality, climate, blue-green space, land use, transportation, morphology, socioeconomic conditions, and heat stress, the ranking in Figure 4 reflects their relative contributions within a common modeling framework. In this dataset, accessibility- and perception-related blue-green space indicators, heat stress, and selected urban-pressure variables contributed more strongly to the prediction of jogging flow than most air-quality, climate, and land-use indicators. Therefore, the following dependence and interaction analyses focus on the higher-ranked variables, while lower-ranked variables are not interpreted as key predictors in this dataset.

The initial predictor set covered multiple dimensions of the urban environment, including blue-green spaces, heat stress, public activity opportunity proxies, air quality, climate, land use, transportation infrastructure, urban morphology, socioeconomic conditions, and environmental ecology. All variables were screened using the same VIF-based procedure before model training. The SHAP interpretation then focused on the top 12 ranked variables, which showed stronger predictive contributions to jogging flow. Other lower-ranked variables, including air-quality indicators and some climate, land-use, and infrastructure indicators, were not interpreted as key predictors in this dataset and were therefore not included in the subsequent detailed dependence, interaction, or PLS-SEM pathway analysis.

### 3.3 Relationships between jogging flow and driving factors

**Figure 5:**
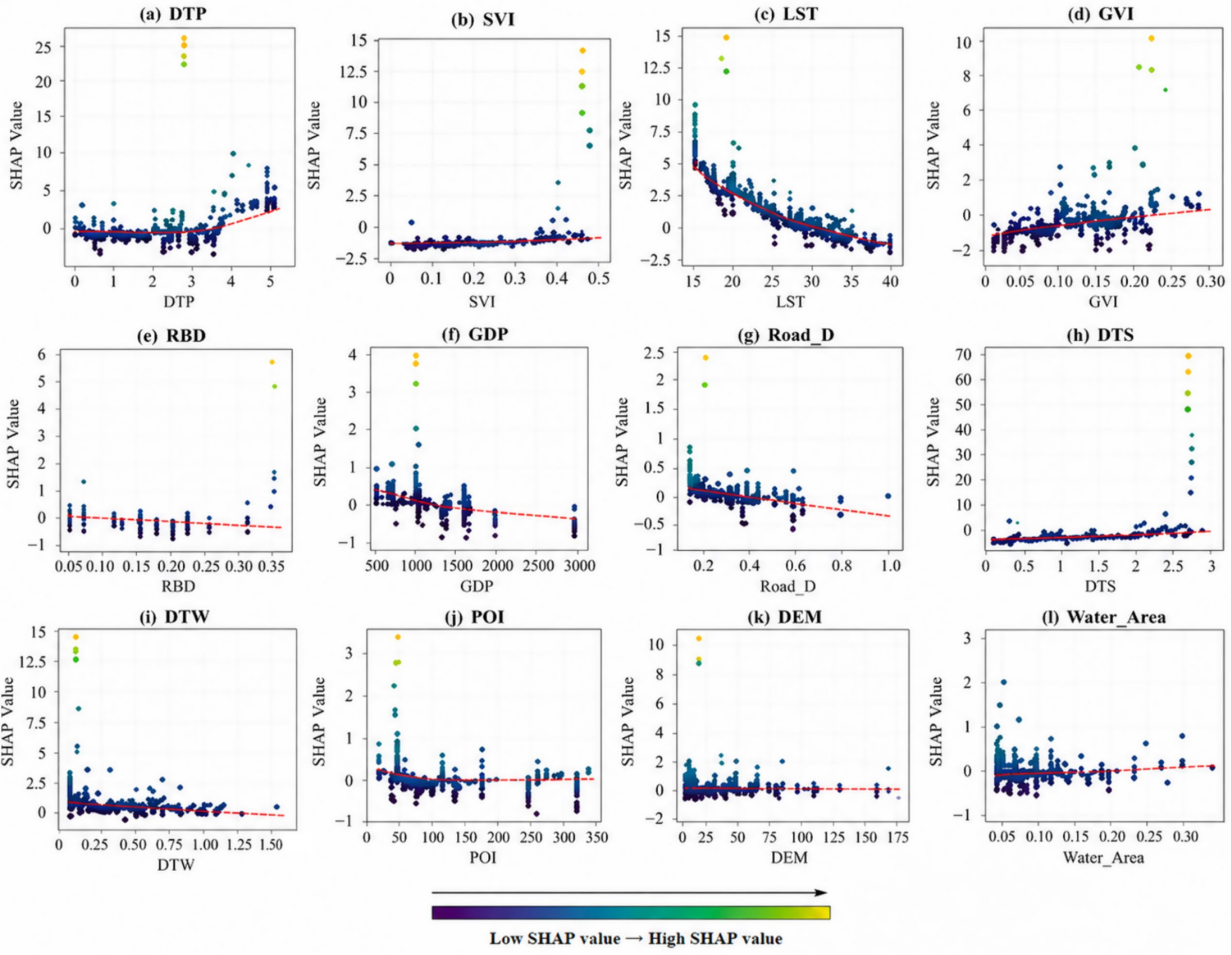
Relationships between jogging flow and driving factors. Panels **(a–l)** correspond to DTP, SVI, LST, GVI, RBD, GDP, Road_D, DTS, DTW, POI, DEM, and Water_Area, respectively. Each panel shows the SHAP dependence pattern of a key predictor. The x-axis represents the value of the predictor, and the y-axis represents its SHAP value for predicted jogging flow. The red dashed line indicates the fitted trend. The point color represents the magnitude of the SHAP value, with darker purple indicating lower SHAP values and yellow indicating higher SHAP values.

Figure 5 shows the nonlinear associations between jogging flow and the top 12 key predictors, including DTP, SVI, LST, GVI, RBD, GDP, Road_D, DTS, DTW, POI, DEM, and Water_Area. Overall, these variables showed nonlinear associations with jogging flow, indicating that the relationships between environmental and urban variables and jogging flow were not simply linear.

Among the blue-green space variables, DTP, SVI, and GVI showed distinct SHAP response patterns. DTP showed a generally positive SHAP pattern at higher values. Because DTP measures the distance from the jogging starting point to the nearest vegetation patch, this pattern should not be interpreted as evidence that being farther from vegetation directly increases jogging flow. Instead, it should be understood as a starting-point-based spatial association, which may differ from the actual green exposure experienced along complete jogging routes. Several unusually high DTP-SHAP values were observed around DTP ≈ 2.5–3. These observations may reflect a small subset of jogging-origin locations where relatively long distance to the nearest vegetation patch coexists with other favorable urban conditions, such as higher residential density, road accessibility, or activity-related spatial opportunities. SVI and GVI showed positive associations with jogging flow across part of their value ranges, suggesting that spatial openness and visible greenery were relevant visual-environment indicators in the prediction of jogging flow. LST showed a negative association with jogging flow. As LST increased, SHAP values tended to decline, indicating that higher surface temperature was associated with lower predicted jogging flow.

For urban pressure and public activity opportunity variables, RBD showed a weak but generally positive association with jogging flow, whereas GDP, Road_D, and POI showed negative SHAP patterns across part of their ranges. DTS and DTW also showed negative associations, indicating that greater distance from sports facilities and water bodies was associated with lower predicted jogging flow. Taken together, Figure 5 shows that jogging flow was associated with a combination of blue-green space accessibility, visual environmental quality, heat-stress conditions, residential density, and urban development intensity. The possible explanations for these patterns are discussed in Section 4.

### 3.4 Interactions among blue-green space variables

**Figure 6:**
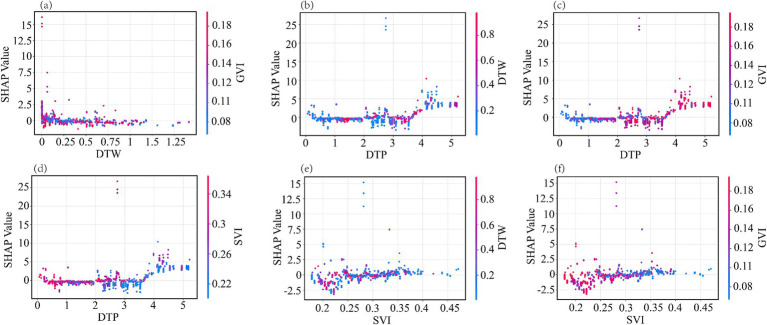
Interactions among blue-green space variables. Panels **(a–f)** show DTW-GVI, DTP-DTW, DTP-GVI, DTP-SVI, SVI-DTW, and SVI-GVI interaction patterns, respectively. Each panel shows the SHAP dependence pattern between two blue-green space variables. The x-axis represents the focal variable, and the y-axis represents the SHAP value of the focal variable for predicted jogging flow. The color gradient indicates the value of the interacting variable shown by the color bar in each panel. The fitted curve shows the overall SHAP response trend.

Figure 6 illustrates several interactions involving blue-green space variables, including DTP-GVI, DTP-SVI, SVI-GVI, and SVI-DTS. Higher DTP combined with higher GVI corresponded to positive SHAP values in part of the sample (Figure 6c). The combination of high SVI and low GVI also corresponded to positive SHAP values (Figure 6f), while high DTP combined with low SVI showed a similar pattern (Figure 6d). These interaction patterns indicate that accessibility, visible greenery, and spatial openness were jointly associated with jogging flow. The possible explanations for these patterns are discussed in Section 4.

These interaction patterns indicate that blue-green space variables did not operate independently; instead, accessibility, visual greenery, and spatial openness were jointly associated with environmental conditions related to health-oriented public-space use.

### 3.5 Interactions between blue-green and other variables

**Figure 7:**
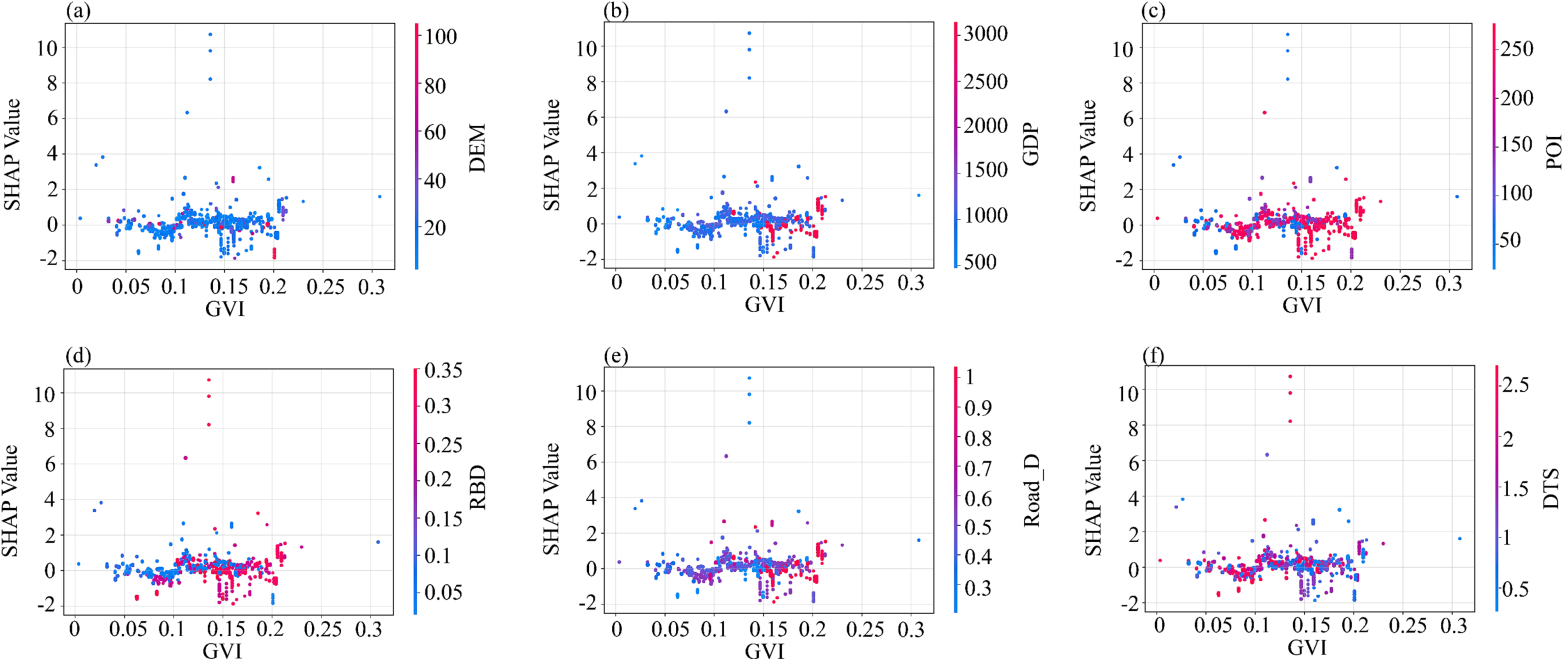
Relationship between GVI and urban variables. Panels **(a–f)** show GVI interacting with DEM, GDP, POI, RBD, Road_D, and DTS, respectively. Each panel shows the SHAP dependence pattern between GVI and an interacting urban variable. The x-axis represents GVI, and the y-axis represents the SHAP value of GVI for predicted jogging flow. The color gradient indicates the value of the interacting urban variable shown by the color bar in each panel. The fitted curve shows the overall SHAP response trend.

**Figure 8:**
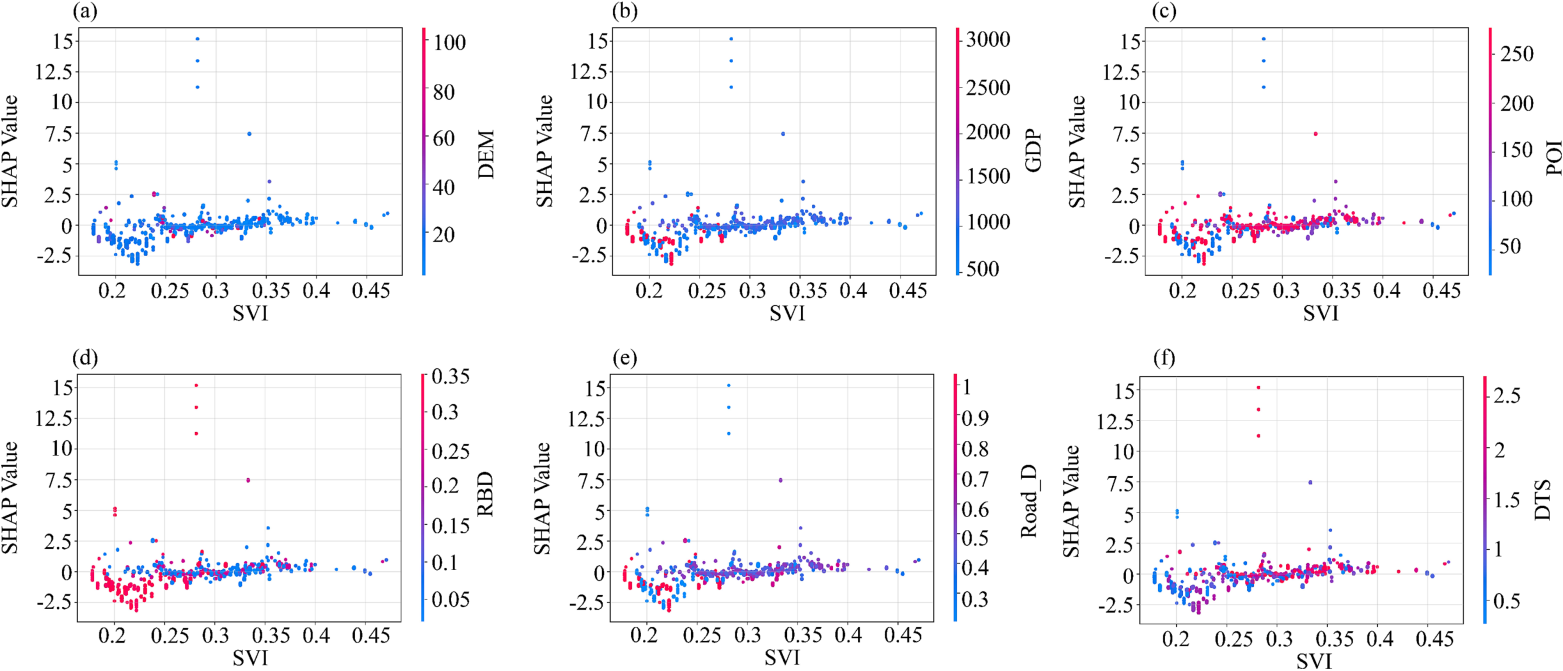
Relationship between SVI and urban variables. Panels **(a–f)** show SVI interacting with DEM, GDP, POI, RBD, Road_D, and DTS, respectively. Each panel shows the SHAP dependence pattern between SVI and an interacting urban variable. The x-axis represents SVI, and the y-axis represents the SHAP value of SVI for predicted jogging flow. The color gradient indicates the value of the interacting urban variable shown by the color bar in each panel. The fitted curve shows the overall SHAP response trend.

**Figure 9:**
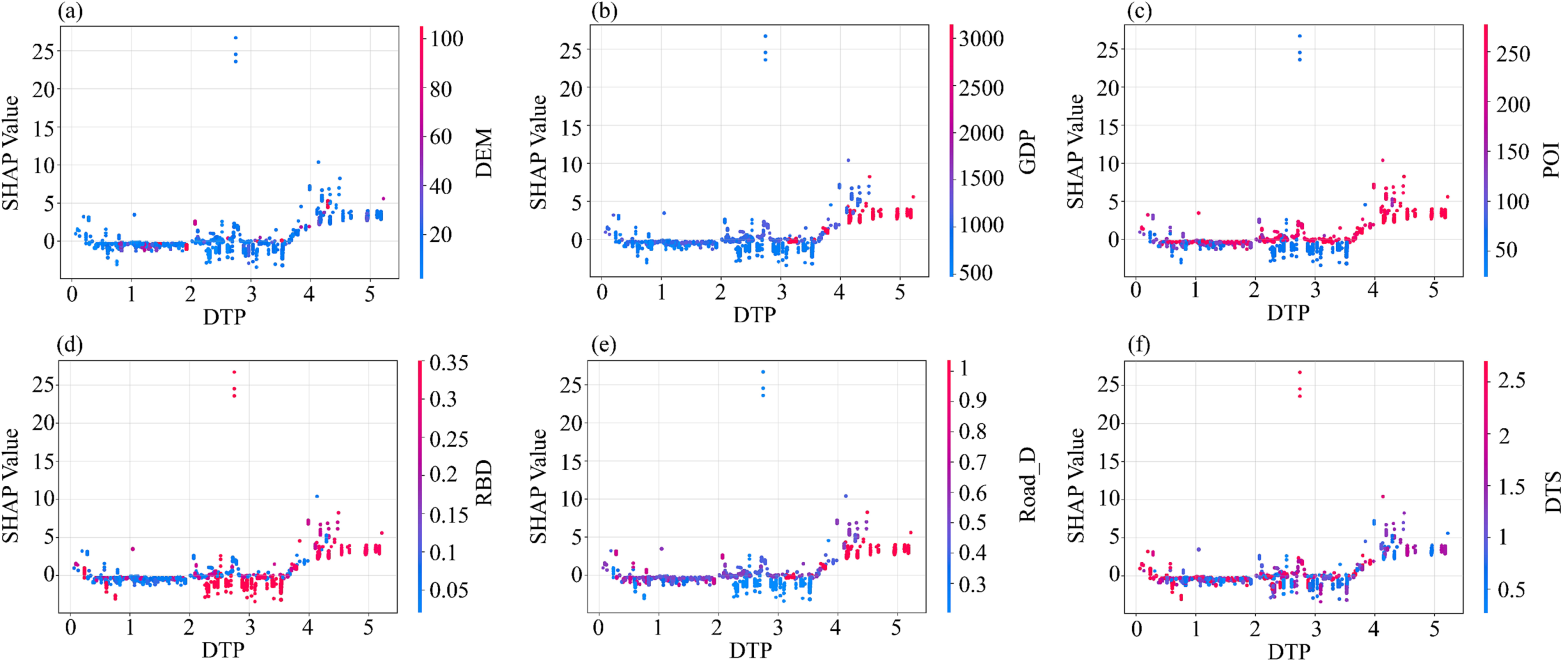
Relationship between DTP and urban variables. Panels **(a–f)** show DTP interacting with DEM, GDP, POI, RBD, Road_D, and DTS, respectively. Each panel shows the SHAP dependence pattern between DTP and an interacting urban variable. The x-axis represents DTP, and the y-axis represents the SHAP value of DTP for predicted jogging flow. The color gradient indicates the value of the interacting urban variable shown by the color bar in each panel. The fitted curve shows the overall SHAP response trend.

**Figure 10:**
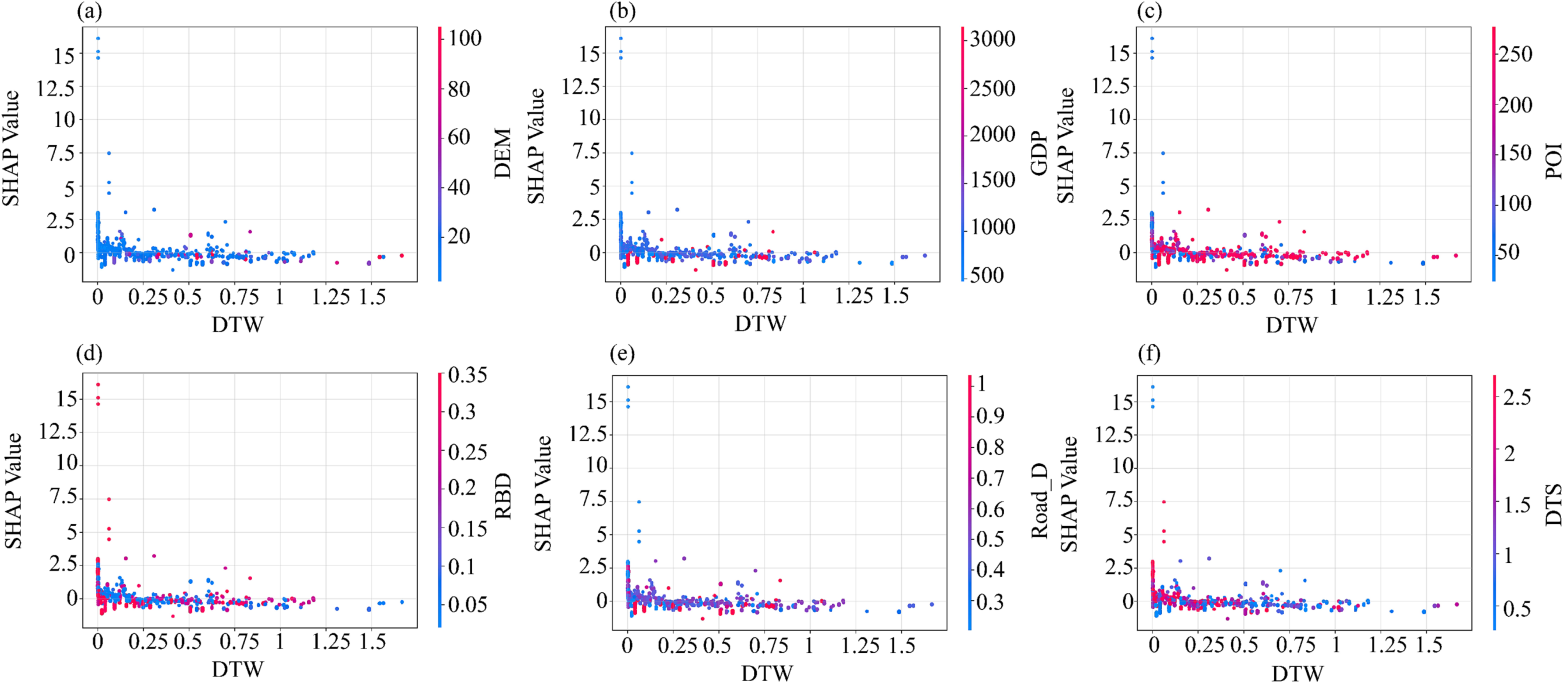
Relationship between DTW and urban variables. Panels **(a–f)** show DTW interacting with DEM, GDP, POI, RBD, Road_D, and DTS, respectively. Each panel shows the SHAP dependence pattern between DTW and an interacting urban variable. The x-axis represents DTW, and the y-axis represents the SHAP value of DTW for predicted jogging flow. The color gradient indicates the value of the interacting urban variable shown by the color bar in each panel. The fitted curve shows the overall SHAP response trend.

Building on the interactions identified among blue-green space variables in the previous section, we further examined whether blue-green space variables also interacted with broader urban pressure and public activity opportunity proxies in relation to jogging flow (Figures 7–10). Overall, GVI, SVI, and DTP showed clearer interaction patterns with selected urban variables than DTW.

For GVI, interactions were observed with RBD and Road_D (Figures 7d,e). However, the direction of SHAP values remained relatively stable across different interaction conditions, suggesting that the association between GVI and jogging flow was not substantially reversed by residential building density or road density. For SVI, the combination of low SVI and high RBD was associated with negative SHAP values (Figure 8d), whereas higher SVI combined with higher DTS corresponded to positive SHAP values in part of the distribution (Figure 8f). These results indicate that spatial openness showed interaction patterns with residential density and sports-facility accessibility. For DTP, interaction patterns were observed with GDP, RBD, and Road_D (Figures 9b,d,e). Higher DTP values combined with higher GDP, RBD, or Road_D were associated with positive SHAP values in part of the sample. Because DTP was calculated from jogging starting points rather than complete jogging routes, this pattern should not be interpreted as evidence that greater distance from vegetation is beneficial for jogging. Instead, it should be understood as a starting-point-based spatial association that may reflect a mismatch between jogging origins and nearby vegetation supply. For DTW, no clear interaction pattern was observed across the selected urban variables, suggesting that its association with jogging flow was relatively independent or localized compared with GVI, SVI, and DTP.

Overall, Figures 7–10 show that blue-green space variables interacted differently with urban pressure and public activity opportunity proxies. The possible explanations for these interaction patterns are further discussed in Section 4.

### 3.6 PLS-SEM pathway associations related to jogging flow

**Figure 11:**
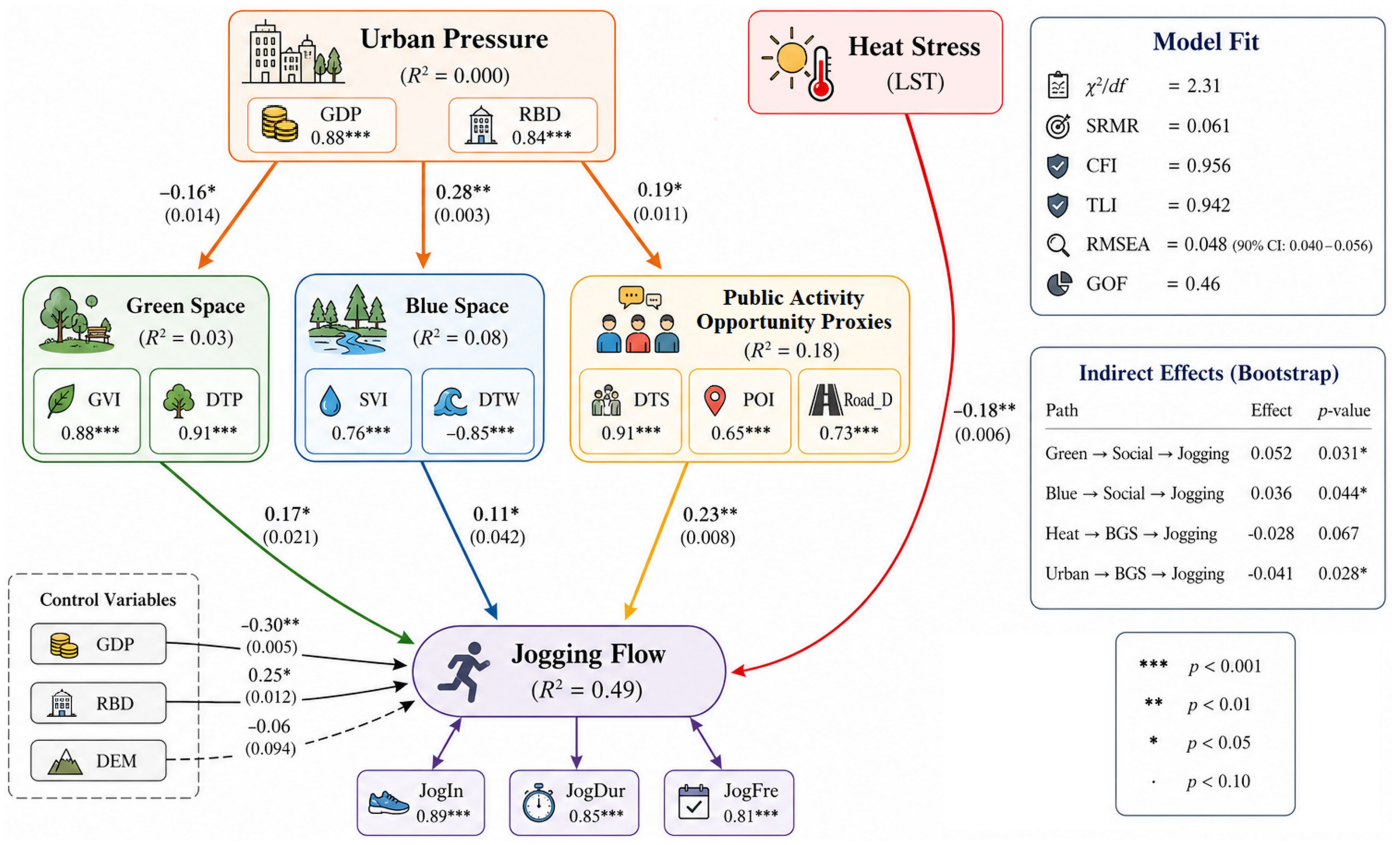
PLS-SEM pathway associations related to jogging flow.

Building on the machine-learning results, PLS-SEM was further used to examine pathway associations among urban pressure, heat stress, blue-green spaces, public activity opportunity proxies, and jogging flow (Figure 11). The model showed an acceptable overall explanatory performance, with a GOF value of 0.46. The *R*^2^ value for jogging flow was 0.49, indicating that the model explained a moderate proportion of the variation in jogging flow. The detailed PLS-SEM results, including model-level indicators, direct pathways, and indirect pathways, are summarized in Table 5.

**Table 5:** Summary of PLS-SEM results.

Panel	Pathway/indicator	Estimate	*p*-value	Significance
Model fit	GOF	0.46	\	\
Explanatory power	*R*^2^ for jogging flow	0.49	\	\
Direct effect	Green space → jogging flow	0.17	0.021	Significant
	Blue space → jogging flow	0.11	0.042	Significant
	Public activity opportunity proxies → jogging flow	0.23	0.008	Significant
	Heat stress → jogging flow	−0.18	0.006	Significant
	Urban pressure → green space	−0.16	0.014	Significant
	Urban pressure → blue space	0.28	0.003	Significant
	Urban pressure → public activity opportunity proxies	0.19	0.011	Significant
Indirect effect	Green space → public activity opportunity proxies → jogging flow	0.052	0.031	significant
	Blue space → public activity opportunity proxies → jogging flow	0.036	0.044	significant
	Urban pressure → BGS-related pathways → jogging flow	−0.041	0.028	Significant
	Heat stress → BGS-related pathways → jogging flow	−0.028	0.067	Marginal

The significance of the path coefficients was assessed using bootstrapping in the PLS-SEM procedure. The direct pathway estimates were positive for green space (*β* = 0.17, *p* = 0.021), blue space (*β* = 0.11, *p* = 0.042), and public activity opportunity proxies (*β* = 0.23, *p* = 0.008) in relation to jogging flow. By contrast, heat stress showed a negative direct pathway estimate (*β* = −0.18, *p* = 0.006). These results indicate that jogging flow was higher in areas with stronger blue-green space conditions and more favorable activity-related spatial conditions, but lower under stronger heat-stress conditions. In addition, Road_D and activity-related variables were mainly connected to jogging flow through indirect pathways. Specifically, Road_D showed a positive pathway estimate with blue-space variables (*β* = 0.30), while public activity opportunity proxies showed a positive pathway estimate with green-space variables (*β* = 0.06).

Urban pressure showed mixed pathway estimates for the intermediate constructs. It was negative for green space (*β* = −0.16, *p* = 0.014), but positive for blue space (*β* = 0.28, *p* = 0.003) and public activity opportunity proxies (*β* = 0.19, *p* = 0.011). This pattern suggests that urban development pressure was linked to weaker green-space conditions but denser blue-space-related and activity-related spatial resources in more developed urban areas.

The mediation results showed that green space had a significant positive indirect association with jogging flow through public activity opportunity proxies (indirect effect = 0.052, *p* = 0.031). Blue space also showed a significant positive indirect association through public activity opportunity proxies (indirect effect = 0.036, *p* = 0.044). Urban pressure showed a significant indirect association with jogging flow through BGS-related pathways (indirect effect = −0.041, *p* = 0.028). The indirect association between heat stress and jogging flow through BGS-related pathways was negative but marginally significant (indirect effect = −0.028, *p* = 0.067).

Overall, the PLS-SEM results indicate that jogging flow was directly associated with green space, blue space, public activity opportunity proxies, and heat stress, and was also indirectly associated with green space, blue space, urban pressure, and heat stress through selected pathway structures. These pathway results provide the empirical basis for the discussion in Section 4.

## 4 Discussion

### 4.1 Heat stress and jogging flow

The negative association between LST and jogging flow indicates that heat-related environmental conditions are closely linked to health-supportive public-space use (33). Among the key variables identified by the SHAP analysis, LST ranked after DTP and SVI and before GVI, suggesting that heat stress was not a peripheral factor in the prediction of jogging flow. In hot and humid cities such as Fuzhou, higher surface temperature may correspond to lower recorded jogging flow, highlighting the need to consider thermal conditions when evaluating public-space usability.

This finding is consistent with previous studies showing that thermal exposure can reduce the attractiveness and usability of outdoor spaces for physical activity, particularly in warm urban contexts (19, 20). However, LST should be interpreted as a surface-temperature-based proxy for urban heat stress rather than a direct measure of human thermal comfort. Actual thermal experience during jogging may also depend on route-level shade, wind, time of day, humidity, and individual adaptation.

This heat-stress finding also clarifies why BGS matter beyond their recreational function. Vegetation, street greenery, waterfront spaces, and open public spaces may provide shade, visual relief, and cooler settings, which are relevant to maintaining outdoor activity under heat-related urban stress.

### 4.2 Visual and accessibility dimensions of blue-green spaces

The results show that blue-green spaces were associated with jogging flow through both visual and accessibility-related dimensions. Among the key predictors identified by SHAP, GVI, SVI, DTP, and DTW represented different aspects of visible greenery, spatial openness, vegetation accessibility, and blue-space accessibility. This suggests that jogging flow was not only related to the presence of green or blue spaces, but also to how these spaces were perceived, accessed, and embedded in the surrounding urban environment.

GVI and SVI were among the most important predictors of jogging flow, indicating the importance of street-level visual conditions. GVI reflects visible greenery along streets or paths, which may be more directly related to jogging experience than large-area green-space indicators alone. Because joggers experience the urban environment at eye level, visible greenery may be linked to visual comfort and perceived environmental quality. SVI provides a complementary dimension by capturing spatial openness, which may correspond to less enclosed streetscapes and clearer route legibility. These results are consistent with previous studies showing that park access, greenways, water bodies, and restorative green-space exposure are associated with outdoor physical activity and psychological benefits (10, 34–37).

Accessibility-related variables further clarify how blue-green spaces were linked to jogging flow. DTS and DTW showed negative associations with jogging flow, indicating that greater distance from sports facilities and water bodies was associated with lower predicted jogging flow. This pattern suggests that nearby activity-supportive destinations and blue-space environments may correspond to lower spatial costs for jogging and may help explain why some locations showed higher jogging-origin intensity. This interpretation is consistent with studies showing that green-space accessibility, supply–demand balance, and urban morphological context are closely associated with physical activity participation and environmental exposure (38–41).

The SEM results further support a combined visual-accessibility interpretation. Green space and blue space were directly associated with jogging flow, and both also showed indirect associations through public activity opportunity proxies. These findings suggest that BGS were linked to jogging flow not only through environmental quality, but also through their connection with activity-related spatial conditions such as destinations, sports facilities, and accessible public-space nodes. Therefore, the association between BGS and jogging flow should be understood as a combined visual-accessibility pathway rather than a single greenness-based relationship.

### 4.3 Urban pressure and spatial mismatch in jogging-supportive environments

The positive SHAP pattern of DTP requires careful interpretation. Since DTP measures the distance from jogging starting points to the nearest vegetation patch, it should not be interpreted as evidence that being farther from vegetation is beneficial for jogging. Instead, this pattern is more likely to reflect the spatial organization of jogging origins in Fuzhou. Some recorded jogging flows may originate from dense residential or commercial areas where nearby vegetation is limited, while the actual jogging routes may extend toward larger parks, waterfront corridors, or other activity-supportive environments.

This interpretation is supported by the interaction results between DTP and GDP, RBD, and Road_D. Higher DTP values combined with higher GDP, residential building density, or road density were associated with positive SHAP values in part of the sample. These patterns suggest that jogging demand may be concentrated in more developed and densely built areas, even when nearby vegetation supply is insufficient. Rather than indicating a preference for low-greenness environments, the DTP result may reveal a spatial mismatch between jogging demand and nearby green-space accessibility. This interpretation is also relevant to the few high DTP-SHAP observations. Previous jogging studies have shown that distance-based built-environment variables may exhibit nonlinear or U-shaped associations with jogging behavior, partly because jogging origins and destinations do not always coincide with the actual activity environment (42, 43). For example, some joggers may start or end their activities in residential or workplace areas but run toward parks, tracks, waterfronts, or other suitable routes. In this study, the high DTP-SHAP observations may similarly indicate a localized mismatch between jogging origins and nearby vegetation supply, rather than a general preference for low-greenness environments.

When placed in the broader urban context, the interaction patterns point to a potential tension between high-density development and jogging-supportive environmental quality. High residential density may indicate a larger potential population of joggers, but it can also be associated with greater competition for public-space resources. Similarly, dense road networks and commercial development may improve urban accessibility while also increasing traffic disturbance, route interruptions, and environmental pressure. This helps explain why some urban-pressure variables showed negative or mixed associations with jogging flow. This pattern is broadly consistent with earlier findings showing that rapid urban expansion can alter green-space structure and accessibility (44, 45), and with studies emphasizing the uneven distribution of urban green-space benefits (46).

The DTP result also highlights a methodological issue. Because DTP was measured at jogging starting points, it captures vegetation accessibility around jogging origins rather than full-route green exposure. This distinction helps explain why high-DTP origins may still be connected to greener destinations along actual jogging routes.

Taken together, the findings contribute to a more nuanced understanding of outdoor jogging behavior. Jogging flow is not only associated with attractive blue-green environments, but also with the relationship between activity demand and environmental supply in dense urban settings. In this sense, the study suggests that jogging-supportive planning should pay attention to high-density areas where residents may have strong demand for outdoor activity but limited nearby access to vegetation or comfortable public spaces.

### 4.4 Planning implications

Based on the associations identified for GVI, SVI, spatial accessibility, heat stress, and public activity opportunity proxies, the findings offer several implications for health-oriented and resilience-oriented public-space planning.

First, heat-sensitive public-space planning should be prioritized in areas with high LST. Shaded jogging corridors, street trees, pocket parks, and waterfront routes could be strengthened as heat-sensitive design measures for improving the usability of outdoor activity spaces during hot periods.

Second, street-level visual quality should be treated as a key dimension of jogging-supportive urban design. Urban renewal strategies can improve visible greenery through sidewalk tree planting, vertical greening, and micro-scale street greening, especially in dense built-up areas where large new parks are difficult to provide.

Third, SVI should be considered together with greenery indicators, because spatial openness may correspond to more legible and less enclosed jogging environments. Urban design should consider street height-to-width ratios, visual corridors, and building enclosure effects, particularly in high-density residential areas.

Fourth, blue-green spaces should be integrated with activity-related spatial conditions, including sports facilities, rest areas, accessible public nodes, and continuous routes. Consistent with the SEM results, blue-green planning should avoid treating green and blue spaces as isolated landscape resources and should instead connect them with everyday activity networks.

Finally, the DTP and urban-pressure interaction results suggest that planning should pay closer attention to high-density neighborhoods with limited nearby vegetation. To address potential supply–demand mismatch, small-scale green interventions such as pocket parks, street greening, and neighborhood-level jogging corridors may improve access to nearby nature and provide more favorable conditions for health-supportive public-space use under urban stress (47, 48).

### 4.5 Limitations

This study has several limitations. First, the jogging data were derived from Keep, a crowdsourced fitness-tracking platform, and therefore may not represent the general population. Keep users are likely to be more physically active, health-conscious, younger, and more familiar with mobile technologies than non-users. In addition, the dataset did not include individual-level demographic and socioeconomic information, such as age, income, occupation, and education. As a result, this study could not examine how the associations between blue-green spaces, heat stress, and jogging flow vary across different population groups. The observed jogging flow should therefore be interpreted as the spatial distribution of recorded activities among active app users rather than as the overall physical-activity pattern of all residents.

Second, this study used jogging starting points as the spatial matching unit. This approach provided a consistent spatial reference for linking jogging records with multi-source environmental variables, but it could not fully capture environmental exposure along complete jogging routes. Future studies should incorporate full trajectory data where available to compare origin-based, route-based, and destination-based exposure measures.

Third, LST was used as a proxy for urban heat stress. Although LST is useful for characterizing surface thermal environments and urban heat patterns, it cannot fully represent human thermal comfort, perceived heat stress, or microclimatic exposure during jogging. Future research could combine LST with air temperature, humidity, wind speed, shade, mean radiant temperature, or wearable-sensor data to better measure route-level thermal exposure.

Fourth, because the data were spatially referenced, potential spatial autocorrelation may affect the estimated associations. Although this study focused on predictive associations rather than spatial causal effects, future work could incorporate spatial machine learning, geographically weighted models, spatial SEM, or spatial cross-validation to more explicitly account for spatial dependence.

Finally, the SEM model should be interpreted as an exploratory pathway model based on cross-sectional observational data. The pathways identified in this study represent statistical associations rather than causal mechanisms. Future studies using longitudinal data, natural experiments, or before-and-after evaluations of blue-green infrastructure interventions could provide stronger evidence on how changes in blue-green space conditions are linked to jogging behavior.

## 5 Conclusion

This study integrated machine learning and SEM to examine how blue-green spaces (BGS), heat stress, urban pressure, and public activity opportunity proxies were statistically associated with jogging flow in Fuzhou. The results showed that CatBoost achieved the best predictive performance among the tested machine learning models, and identified DTP, SVI, LST, and GVI as the principal factors associated with jogging flow. Specifically, visible greenery and spatial openness were positively associated with jogging flow, whereas higher LST was associated with lower predicted jogging flow. The positive SHAP pattern of DTP should be interpreted cautiously, because DTP was measured from jogging starting points rather than complete jogging routes.

The pathway analysis further suggested a possible association structure linking blue-green spaces, public activity opportunity proxies, heat stress, urban pressure, and jogging flow. Green space, blue space, and public activity opportunity proxies were positively associated with jogging flow, while heat stress showed a negative direct association. In addition, green and blue spaces showed indirect associations with jogging flow through public activity opportunity proxies. These results indicate that blue-green environments may be linked to jogging flow not only through their environmental qualities, but also through their connections with activity-supportive spatial conditions. However, these pathways should be interpreted as exploratory statistical associations rather than causal mechanisms.

Overall, the findings suggest that jogging flow is associated with visual greenery, spatial openness, blue-green space accessibility, public activity opportunity proxies, and surface thermal conditions. These insights are relevant to health-oriented and resilience-oriented public-space planning in high-density cities facing heat stress and uneven blue-green space accessibility. However, the study does not directly measure community resilience, social cohesion, or individual-level thermal exposure. Its contribution lies in identifying possible spatial and environmental pathways linking blue-green environments to health-supportive public-space use.

## Data Availability

The datasets presented in this article are not readily available because requests to access the datasets should be directed to the corresponding author, sunjian@fzu.edu.cn.
